# Integrated physiological, transcriptomic, and metabolomic analyses of drought stress alleviation in *Ehretia macrophylla* Wall. seedlings by SiO_2_ NPs (silica nanoparticles)

**DOI:** 10.3389/fpls.2024.1260140

**Published:** 2024-02-02

**Authors:** Minghui Chen, Si-qian Jiao, Lihua Xie, Xining Geng, Shuaizheng Qi, Jianmin Fan, Shiping Cheng, Jiang Shi, Xibing Cao

**Affiliations:** ^1^ Pingdingshan University, Henan Province Key Laboratory of Germplasm Innovation and Utilization of Eco-economic Woody Plant, Pingdingshan, China; ^2^ College of Agriculture, Henan University of Science and Technology, Luoyang, China; ^3^ College of Forestry, Henan Agricultural University, Zhengzhou, Henan, China

**Keywords:** *Ehretia macrophylla* Wall., silicon dioxide nanoparticles, drought stress, transcriptomic, metabolomic

## Abstract

With environmental problems such as climate global warming, drought has become one of the major stress factors, because it severely affects the plant growth and development. Silicon dioxide nanoparticles (SiO_2_ NPs) are crucial for mitigating abiotic stresses suffered by plants in unfavorable environmental conditions and further promoting plant growth, such as drought. This study aimed to investigate the effect of different concentrations of SiO_2_ NPs on the growth of the *Ehretia macrophylla* Wall. seedlings under severe drought stress (water content in soil, 30–35%). The treatment was started by starting spraying different concentrations of SiO2 NPs on seedlings of Ehretia macrophyla, which were consistently under normal and severe drought conditions (soil moisture content 30-35%), respectively, at the seedling stage, followed by physiological and biochemical measurements, transcriptomics and metabolomics analyses. SiO_2_ NPs (100 mg·L^−1^) treatment reduced malondialdehyde and hydrogen peroxide content and enhanced the activity of antioxidant enzymes under drought stress. Transcriptomic analysis showed that 1451 differentially expressed genes (DEGs) in the leaves of *E*. *macrophylla* seedlings were regulated by SiO_2_ NPs under drought stress, and these genes mainly participate in auxin signal transduction and mitogen-activated protein kinase signaling pathways. This study also found that the metabolism of fatty acids and α-linolenic acids may play a key role in the enhancement of drought tolerance in SiO_2_ NP-treated *E. macrophylla* seedlings. Metabolomics studies indicated that the accumulation level of secondary metabolites related to drought tolerance was higher after SiO_2_ NPs treatment. This study revealed insights into the physiological mechanisms induced by SiO_2_ NPs for enhancing the drought tolerance of plants.

## 1 Introduction

Drought is one of the major stress factors affecting the growth and development of plants (Batool et al., 2020; Seleiman et al., 2021; Roy et al., 2021). Drought incidences have increased globally due to factors, such as global warming, low precipitation, land exploitation, and over-utilization of water resources (You et al., 2019; Zhu et al., 2021). In several parts of the world, drought represents the main limiting factor for agricultural production and ecological restoration (Seleiman et al., 2021). Therefore, the adaptation of plants to drought stress and associated drought resistance mechanisms have attracted great attention in various fields, including environmental science, ecology, and genetics (Wan et al., 2022). However, the evaluation of drought resistance and associated mechanisms is a highly complex process due to intricate interactions of genetic and environmental factors (Gupta et al., 2020). Plants adapt to drought through a series of morphological, physiological, biochemical, metabolic, and molecular changes (Baozhu et al., 2022; Thanmalagan et al., 2022). For instance, under water-shortage stress, Norway spruce seedlings reduce photosynthesis, decreasing their growth and biomass to regulate the needle leaf osmotic potential, relative water content, and levels of primary metabolites (Jamnická et al., 2019). In potato seedlings, drought resistance is mediated by increased levels of polyamines (PAs), abscisic acid (ABA), proline (Pro), and soluble sugars in leaves and the activity of antioxidant enzymes (Zhang et al., 2018). Further, when *Prunus salicina* leaves were under drought stress, a sharp decrease was observed in water osmotic potentials and relative water content of *P. salicina* leaves, which decrease significantly in gaseous exchange processes (Hajlaoui et al., 2022). Another study have shown that the plants mitigate the water-deficiency damages by reducing Gs, Pn, Fv/Fm, and chlorophyll index (Ammar et al., 2020).

Plants improve their drought resistance by changing cellular, physiological, and biochemical processes, and these changes are governed by plant-related metabolites and the expression pattern of different associated genes (Al-Ashkar et al., 2021). Therefore, it is important to understand the molecular mechanism of plant drought resistance to achieve further improvements in drought resistance and to breed drought-resistant varieties. In recent years, significant progress has been made in conducting transcriptomic and metabolomic studies to under drought resistance in plants. Zhao et al. (Zhao et al., 2022) discovered candidate genes (*PP2C*, *PYL, ABF, WRKY33, P5CS, GPX*, *GST*, *CAT*, and *SOD1*), which may control the drought resistance in *Sophora davidii*; oxidized glutathione, ABA, and phenylalanine were found to be associated with drought resistance in this plant. Sun et al. (Sun et al., 2022) exposed *Tamarix taklamakanensis* to drought stress and identified differentially expressed genes (DEGs) involved in the metabolism of tryptophan and α-linolenic acids, biosynthesis of flavonoids and phenylpropanoid compounds, and mitogen-activated protein kinase (MAPK) signaling pathway. In another study, Hu et al. (Hu et al., 2022b) the structural gene expression profile of the flavonoid pathway in *Zanthoxylum bungeanum* leaves and also identified 231 flavonoid compounds under drought stress conditions. Furthermore, three genes (*FBA3*, *DELTA-OAT*, and *PROC*) and 15 transcription factors, related to the biosynthesis of amino acids, were identified in *Z. bungeanum* leaves under drought conditions (Hu et al., 2022a). Transcription and metabolic level changes under drought conditions have been studied in *Seriphidium transiliense* (Liu et al., 2022), *Panicum miliaceum* (Yuan et al., 2022), *Carthamus tinctorius* (Wei et al., 2020), and sweet sorghum (Wang et al., 2022) and stress-related genes and key metabolic pathways and metabolites were analyzed.

Nanoparticles (NPs) range, in size, from 1 to 100 nm, or even smaller particles or atomic aggregates (Khan et al., 2019; Seleiman et al., 2020a; Seleiman et al., 2023a). Previously, it has been postulated that nano fertilizers may be more effective in enhancing plant nutrition and protecting plants from environmental stress compared with regular fertilizers (Seleiman et al., 2020b; Verma et al., 2021; Elshayb et al., 2022a; Elshayb et al., 2022b; Alhammad et al., 2023). In recent times, silicon-based fertilizers have received increasing attention for their ability to alleviate the adverse effects of drought stress in plants (Malik et al., 2021). It has been reported that silicon fertilizers can alleviate drought stress by improving the antioxidant defense systems and photosynthetic metabolism (Johnson et al., 2022; Xu et al., 2022), maintaining intracellular homeostasis (Bhardwaj and Kapoor, 2021), tuning the auxin levels (Mir et al., 2022), and regulating the homeostasis of the oxidation of nitroso-compounds (Bhardwaj et al., 2022). The bioavailability of silicon fertilizers is less, however, NP-based silicon fertilizers were more likely to penetrate into the leaf cells and play a more direct function (Biju et al., 2021), leading to the usage of nanoparticles (NPs) could be an important approach to alleviate soil salt stress (Badawy et al., 2021).

Silicon dioxide (SiO_2_) NPs are single particles of silica, with a diameter of <100 nm, which has been widely used in the world in recent years (Whitesides, 2005; Karimi and Mohsenzadeh, 2016; Al-Selwey et al., 2023b). Studies on stress resistance of plants have shown that SiO2 NPs, as an exogenous application, SiO_2_ NPs can promote plant growth under stress, alleviate various stresses, and stress resistance characteristics in plant (Badawy et al., 2021; Seleiman et al., 2023b; Iqbal et al., 2024). For example, SiO_2_ NPs as an exogenous application on the physiological indices, total yield and water use efficiency (WUE) of potato under water deficit conditions (Al-Selwey et al., 2023a; Al-Selwey et al., 2023b). Hence, they have been used to improve plant growth under abiotic stress (Sheikhalipour et al., 2021). For instance, spraying SiO_2_ NPs on the leaves under drought stress can reduce the content of proline, soluble sugars, and ABA, reduce membrane damage, increase the yield and fruit weight of ‘Kalamata’ olive trees, and reduce fruit drop rates (Hassan et al., 2022). SiO_2_ NPs enhanced the ability of wheat plants to resist water scarcity by balancing the production of reactive oxygen species (ROS) and enhancing the antioxidant system under drought stress (Rai-Kalal et al., 2021). Furthermore, when SiO_2_ NPs have combined with plant growth-promoting bacteria (PGPB), the combination increased the concentration of nitrogen, phosphorus, potassium, and silicon in rapeseed and wheat seedlings, decreased the content of malondialdehyde (MDA), and significantly increased the water deficiency tolerance of rapeseed and wheat (Valizadeh-rad et al., 2023).

*E*. *macrophylla* belongs to the Boraginaceae family and produces deciduous and healthy fruits. This tree is widely distributed in China, Japan, Vietnam, and Nepal (Gottschling and Hilger, 2004). Since *E. macrophylla* fruits have antioxidant properties and contain crude bran polysaccharides, they can be used to treat respiratory diseases, lower blood sugar, and regulate colon health (Deng et al., 2020; Xu et al., 2022). Despite several measures, drought stress is significantly affecting the growth and yield of *E. macrophylla*. At present, the role of SiO_2_ NPs on *E*. *macrophylla* under drought conditions is not well studied. Furthermore, the understanding of the potential processes and the specific mechanisms by which SiO_2_ NPs function in major plants is lacking. We hypothesized that SiO_2_ NPs can regulate the antioxidant system in *E*. *macrophylla* and induce the expression of related genes and metabolites, there by stimulating plant growth. To test this idea, SiO_2_ NPs were sprayed on the leaves of *E. macrophylla* under drought stress and these leaves were subjected to physiological, biochemical, transcriptomic, and metabolomic analysis. This study illustrates a detailed understanding of the relationship between SiO_2_ NPs and plants under drought stress and elucidates possible regulatory mechanisms by which SiO_2_ NPs enhance drought resistance, providing new directions for the potential utilization of SiO_2_ NPs.

## 2 Materials and methods

### 2.1 Characterization of commercial SiO2 NPs and preparation of dilutions

SiO_2_ NPs were purchased from Sigma-Aldrich, USA (purity >98%, size <40 nm) and sputtered and coated with gold for approximately 30 s, followed by observation and image acquisition using a scanning electron microscope (SEM; SU8100, Hitachi). SEM micrographs were processed using the Image J software system, and the particle size was calculated. SiO_2_ NPs were diluted in double distilled water (DDW) to prepare solutions of different concentrations (50, 100, 200, 500, and 1000 mg·L^−1^). At the time of use, 0.05% Tween-20 was added to SiO_2_ NP solutions for even spraying of leaves.

### 2.2 Experimental setup

The experiment was conducted in a greenhouse facility at Pingdingshan College in Pingdingshan City, Henan Province, China. Uniformly-sized *E*. *macrophylla* seeds were soaked in distilled water for 72 h and subsequently disinfected with 75% ethanol. The seeds were then sown in plastic pots (21 cm × 25 cm; the matrix soil was a 1:1:1 mixture of peat, vermiculite, and perlite; 1 plant per pot) and allowed to grow under natural conditions for 3 months, with sufficient water supply. Treatment was started when six true leaves were unfolded. Grown seedlings were divided into groups (six plants per group and three biological replicates per group). The CK group contained seedlings sprayed with DDW and cultured under normal water content (water content in soil, 75–80%). The NPs group had seedlings sprayed with different concentrations of SiO_2_ NPs (50, 100, 200, and 500 mg·L^−1^) and cultured under normal water content. The SD group had seedlings grown under severe drought conditions (water content in soil, 30–35%). Treatment groups had seedlings sprayed with different concentrations of SiO_2_ NPs (50, 100, 200, and 500 mg·L^−1^) and cultured under severe drought conditions (water content in soil, 30–35%). In all these groups, for 1–3 consecutive days, SiO_2_ NPs solutions were applied to the leaves, and samples were collected on day 7 (**Supplementary Figure 1**). Soil moisture content was measured according to gravimetric method and monitored daily until the end of experiment using a TZS-IIW Soil Moisture Meter (Zhejiang Top Instrument Co., Ltd, Zhejiang, China). The water content was kept at 30% to 35% of saturated soil water content in the SD group and the NPs-SD group for 7 days. The soil moisture content is expressed as a percentage of the weight of moisture contained in the soil to the weight of the dried soil. The calculation formula is as follows: soil moisture content (weight %) = (original soil weight - dried soil weight)/dried soil weight ×100% = water weight/dried soil weight ×100%. A portion of the samples was stored at -20°C for physiological and biochemical measurements, whereas the other portion of samples was divided into two parts (for transcriptomics and metabolomics), frozen in liquid nitrogen, and stored at -80°C.

### 2.3 Leaf ultrastructure

The second leaf of seedlings from all groups was cut into slices (2-5 mm), fixed with 2.5% (v/v) glutaraldehyde, and dehydrated using a series of different ethanol concentrations (30%, 50%, 70%, and 96%). Subsequently, the samples were permeated into a mixture of acetone and SPI-Pon812 epoxy resin and embedded in a pure resin. The samples were then sliced (70 nm thick sections) using an ultra-thin slicing machine (Leica EM UC6, Leica, Germany) and observed using a transmission electron microscope (HT-7800, Hitachi, Japan).

### 2.4 Determining the content of malondialdehyde and hydrogen peroxide and antioxidant enzyme activities

MDA content was estimated as described by Siddiqui et al. (Siddiqui et al., 2018), whereas the hydrogen peroxide (H_2_O_2_) content was measured using the method described by Patterson et al. (Patterson et al., 1984). The crude extract of antioxidant enzymes was prepared according to the method described by Mrinalini et al. (Srivastava et al., 2018). The 0.5 g *E*. *macrophylla* leaf sample was quickly ground and crushed in liquid nitrogen, and then homogenized in 3 ml pre-cooled extraction buffer (50 mM phosphate buffer, pH 7.8, 0.1 mM ethylenediaminetetraacetate (EDTA), 0.3%(v/v) Triton X-100, 4% polyvinylpyrrolidone (PVP). Immediately centrifuge the reaction mixture at 12,000xg and 4°C for 20min. The supernatant was analyzed for antioxidant enzyme activity. The activity of peroxidase [POD; the guaiacol method (Bestwick et al., 1998)], superoxide dismutase [SOD; quantitative photochemical degradation of nitro blue tetrazolium (Dhindsa and Matowe, 1981)], catalase [CAT; (Chance and Maehly, 1955)], and ascorbic acid peroxidase [APX; (Nakano and Asada, 1981)] was measured using methods described previously.

### 2.5 RNA extraction, library construction, and sequencing

Total RNA was extracted from leaves of seedlings belonging to CK, NP, SD, and NP-SD (treated with 100 mg·L^-1^SiO_2_ NPs) groups using TRIzol Total RNA Extraction Kit (Tiangen, China) and quantitated using NanoDrop 2000 (Thermo Fisher Scientific, USA). The integrity of RNA was evaluated using the RNA Nano 6000 assay kit Bioanalyzer 2100 system (Agilent Technologies, USA). mRNA population with polyA tail was enriched using Oligo (dT) magnetic beads. The resulting mRNA population was randomly interrupted with divalent cations in the NEB fragmentation buffer, followed by the application of the NEB library construction method. After library construction, the preliminary quantification was performed using Qubit2.0 Fluorometer, and the library was diluted to a concentration of 1.5 ng·μL^−1^. The insert size of the library was analyzed using the Agilent 2100 bioanalyzer, and once the insert was found to be of the expected size, quantitative real-time PCR (qRT-PCR) was performed to quantify the effective concentration of the library to ensure the library quality. Finally, Illumina sequencing was performed after pooling different libraries according to the effective concentration and target offline data volume requirements.

### 2.6 Transcriptomic data analysis

Raw sequencing data were filtered to remove sequences with joints, those containing N bases, and those with low quality and subjected to sequencing error rate and GC content distribution analyses. The processed sequences were mapped to the reference genome using HISAT2 software and assembled using StringTie v1.3.1. For each transcriptional region, the FPKM value was calculated to quantify the expression abundance. Based on the comparison results of HISAT2, transcripts were reconstructed using Stringtie, and the expression levels of all genes in each sample were calculated using RSEM.

DESeq2 was used to analyze differential expression among groups (Love et al., 2014). The screening conditions for DEGs were |log2Fold Change|≥1.00, with a false discovery rate (FDR) of <0.05. All DEGs were mapped to Gene Ontology (GO), which is a set of annotated categories related to biological processes, molecular functions, and cellular components of various genes, terms in the GO database (http://www.geneontology.org/), and the number of genes for each term was calculated. The Kyoto Encyclopediaof Genes and Genomes (KEGG) is a resource for advanced functions of biological systems based on biological pathways (http://www.genome.jp/kegg/), and KEGG annotations include genes as well as metabolites. Hence, differentially expressed metabolites (DEMs) were mapped to the KEGG metabolic pathways for pathway and enrichment analyses.

### 2.7 Quantitative real-time PCR validation

qRT-PCR was used to validate RNA-seq data for 9 different genes (three redox genes, four hormone-associated with signal transduction and MAPK signaling pathway genes, and two genes related to the metabolism of fatty acids and α-linolenic acids). First, cDNA was synthesized from total RNA using the PrimeScript RT kit (TaKaRa, Dalian, China). Primers were designed using the Primer-BLAST software on the NCBI website and synthesized by Shanghai Shenggong Biotechnology Co., Ltd. (Shanghai, China) (**Supplementary Table 1**). qPCR was performed on the StepOnePlus real-time fluorescence qPCR system (ABI, Foster City, CA, USA). The expression level of DEGs was calculated using the 2^−ΔΔCt^ method (Livak and Schmittgen, 2001).

### 2.8 Extraction and quantification of metabolites

Samples stored at 4°C were slowly thawed, and 100 mg of the sample was added to a pre-cooled methanol/acetonitrile/water solution (2:2:1, v/v), followed by vortexing. The mixture was sonicated at low temperature for 30 min, incubated at -20°C for 10 min, and centrifuged at 4°C for 20 min at 14000 xg. The resulting supernatant was then vacuum dried and subjected to mass spectrometry. During mass spectrometry analysis, the dried mixture was supplied with 100 μL acetonitrile aqueous solution (acetonitrile: water=1:1, v/v) for dissolving, followed by vortexing and centrifugation at 4°C for 15 min at 14000 xg. Obtained supernatant was filtered through a microporous membrane (pore size 0.22 µm) for ultra-high performance liquid chromatography-mass spectrometry (UPLC-MS/MS) analysis.

Based on the results of orthogonal partial least squares discriminant analysis (OPLS-DA), a combination of *p*-values of Student’s *t*-tests (*p*<0.1) and variable importance projection (VIP) values of the OPLS-DA model (VIP>0.1) was used to screen DEMs. Subsequently, we constructed a metabolic pathway based on the KEGG database.

### 2.9 Statistical analysis

IBM SPSS Statistics (Version.20, IBM., Amonk, New York United States) software was used for conducting a one-way analysis of variance (ANOVA). Further, the Duncan text was used to decipher the statistical significance of the data (*p ≤*0.05).

## 3 Results

### 3.1 Nano characterization

**Figure 1:**
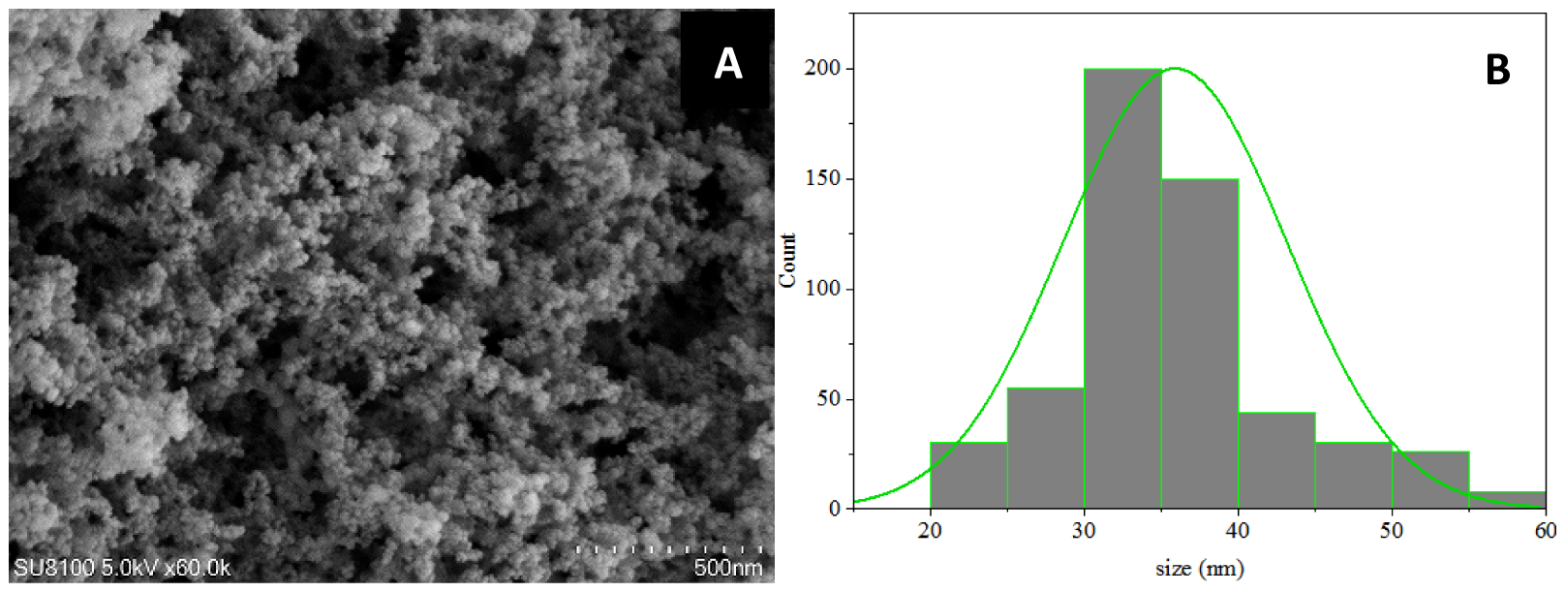
**(A)** Characterization of SiO_2_ NPs using Scanning Electron Microscopy (SEM). Scale bar, 500 nm. **(B)** Particle size distribution based on the SEM image analysis. Averages ± standard deviations.

SEM-based analysis of SiO_2_ NPs demonstrated that SiO_2_ NPs composed of single crystal particles (mainly circular), with a size that ranged from 30 to 40 nm. The average particle size was estimated to be 36 ± 5.3 nm (**Figures 1A, B**).

### 3.2 SiO_2_ NPs promote the growth of *E. macrophylla* seedlings under drought stress

**Figure 2:**
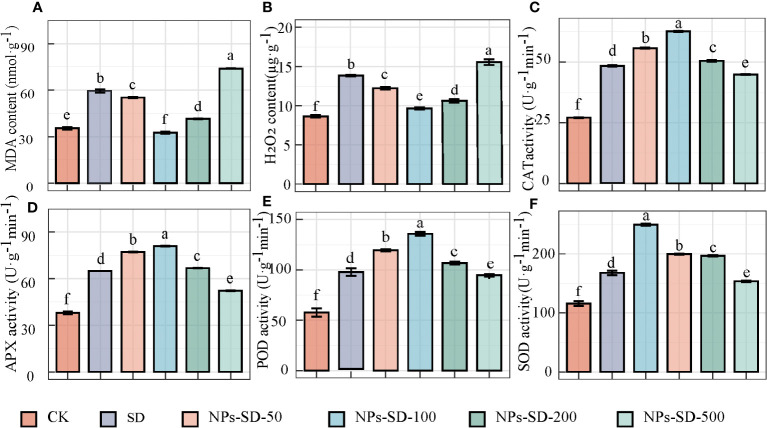
Effects of SiO_2_ NPs on **(A)** malondialdehyde (MDA) content, **(B)** H_2_O_2_ content, **(C)** catalase (CAT) activity, **(D)** Ascorbate peroxidase(APX) activity, **(E)** peroxidase (POD) activity and **(F)** superoxide dismutase (SOD) activity, of *E. macrophylla* seedlings under drought stress. The value represents the average of the 3 biological replicas. Different lowercase letters indicate significant differences among treatments at the same timepoint (p < 0.05). CK, control treatment; SD, severe drought (30%-35% soil water content) treatment; NPs-SD-50、NPs-SD-100、NPs-SD-200 and NPs-SD-500 severe drought seedlings pretreated with 50, 100, 200, or 500 mg·L^−1^ SiO_2_ NPs, respectively.

When *E*. *macrophylla* seedlings under severe drought stress were treated with different concentrations of SiO_2_ NPs (50, 100, 200, and 500 mg·L^−1^), compared with the CK group, the content of MDA and H_2_O_2_ in the SD group was found to be higher by 67.83% and 60.07%, respectively. Compared with the SD group, the content of MDA in *E*. *macrophylla* seedlings treated with 50, 100, and 200 mg·L^−1^ SiO_2_ NPs decreased by 7.15%, 45.21%, and 30.34%, respectively, whereas the content of H_2_O_2_ decreased by 11.64%, 30.37%, and 23.28%, respectively. Furthermore, in comparison to the CK group, the content of MDA and H_2_O_2_ increased by 24.26% and 12.44%, respectively, in the SD group treated with 500 mg·L^−1^ SiO_2_ NPs (**Figures 2A, B**).

Significant differences were observed in the activity of CAT, APX, POD, and SOD under different treatment conditions. The activities of CAT, APX, POD, and SOD in the SD group increased by 78.15%, 70.59%, 67.75%, and 44.50%, respectively, compared with those in the CK group. When seedlings were treated with SiO_2_ NPs (at 50, 100, and 200 mg·L^−1^), in comparison to the SD group, the activity of CAT, APX, POD, and SOD increased by 15.49%, 29.81%, and 4.62%, 18.98%, 24.63%, and 2.94%, 24.79%, 43.07%, and 8.30%, and 18.98%, 48.75%, and 17.24%, respectively. However, when treated at 500 mg·L^−1^ SiO_2_ NPs, the CAT, APX, POD, and SOD activities decreased by 6.94%, 19.43%, 1.61%, and 8.41%, respectively compared to those in the SD group (**Figures 2C–F**).

Hence, although 100 mg·L^−1^ SiO_2_ NPs imparted a positive effect on the growth and development of *E*. *macrophylla* seedlings under drought stress, toxicity was observed at a concentration of 500 mg·L^−1^. Therefore, samples from CK, NPs (treated with 100 mg·L^−1^ SiO_2_ NPs), SD (severe drought), and NPs-SD (100 mg·L^−1^ SiO_2_ NPs + SD) groups were selected for transcriptomics and metabolomics analyses.

### 3.3 Effects of SiO_2_ NPs on the ultrastructure of *E. macrophylla* leaf cell under drought stress

**Figure 3:**
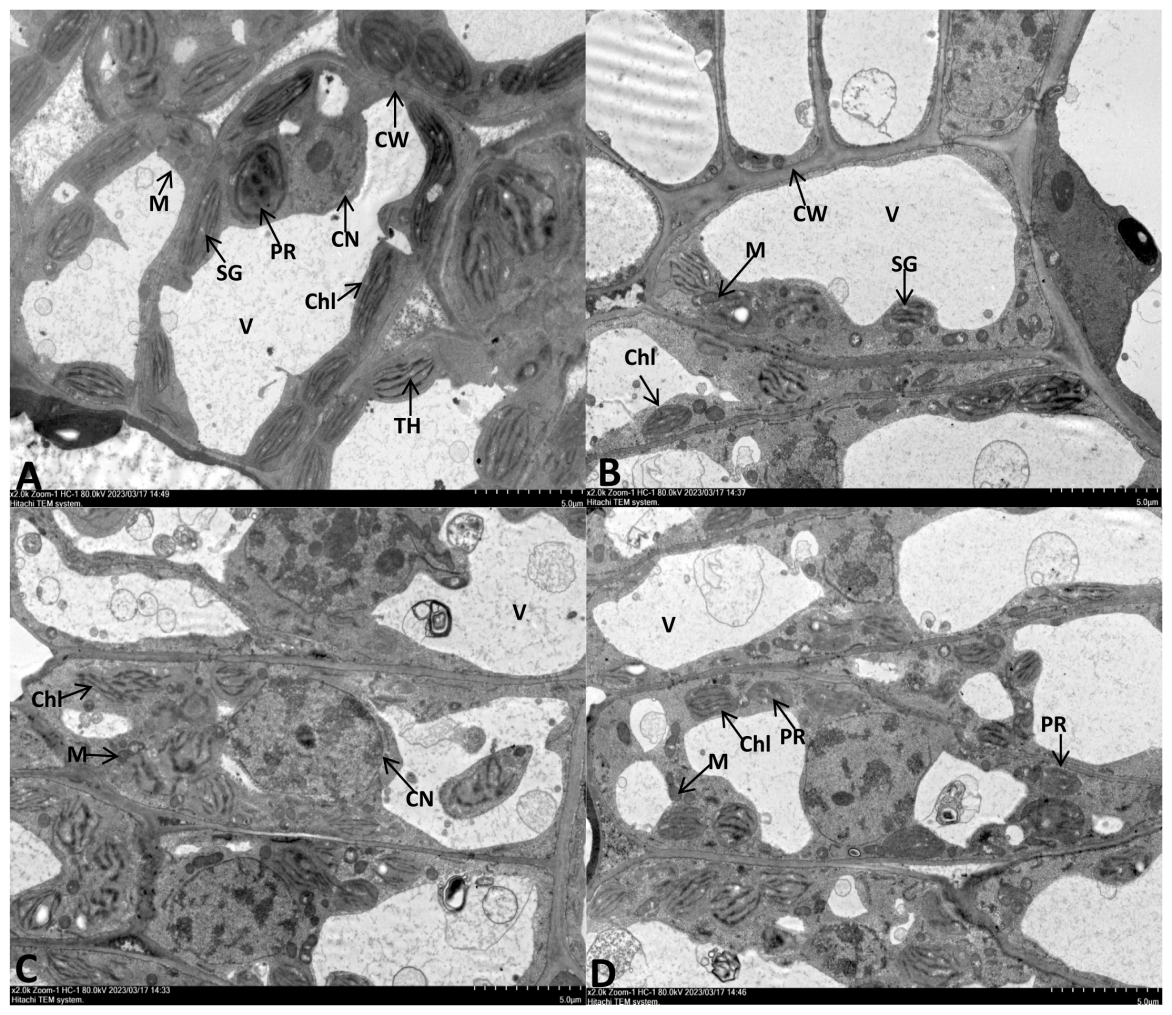
TEM observation of *E. macrophylla* leaf cell under different trentment. **(A)** Control plant. **(B)** 100 mg·L^−1^SiO_2_ NPs-treated. **(C)** Severe drought (SD)- treated. **(D)** 100 mg·L^−1^SiO_2_ NPs-SD-treated. CW cell wall, Chl chloroplast, V vacuole, M mitochondria, SG starch grains, TH thylakoid, PR proplastid, CN cell nucleus.

The leaf cell ultrastructure of the CK and NPs groups were normal, with no cytoplasmic wall separation, intact nucleus, and visible nuclear membrane. The chloroplasts were oval or spindle-shaped, with well-developed and arranged cristae and plastoglobuli of thylakoids. Furthermore, no damages were observed in the mitochondria and the inner cristae. The NPS-treated group had more rounded chloroplasts and slightly reduced matrix electron density. (**Figures 3A, B**). Under drought stress, chloroplast expansion was observed, with the separation of the cytoplasmic wall. At the same time, the chloroplast membrane detached from the thylakoid structure, with a loose granular lamellar structure. The mitochondria were also observed to have damages; the outer membrane was unclear, with gradual dissolving or even rupturing of the outer membrane. The thylakoid membrane also lost its integrity, showing an abnormal shape and obvious signs of damage (**Figure 3C**).

In the NPs-SD (100 mg·L^−1^ SiO_2_ NPs) treated group, chloroplasts of the leaf cells showed spindle-shaped morphology, with intact membrane and clear particle layer structures but beginning to show deformation. The nucleus was not damaged, but the nuclear membrane was somewhat unclear, and the degree of nucleolus aggregation was higher than that of the CK group. Furthermore, in the NPs-SD (100 mg·L^−1^ SiO_2_ NPs) treated group, the number of mitochondria was higher than that of the SD group, with a clear outer membrane. The number of protoplasts also increased, with a decrease in the level of vacuolization (**Figure 3D**). Hence, SiO_2_ NPs pre-treatment decreased the level of leaf damage under drought stress.

### 3.4 RNA-sequencing analysis

To understand the molecular mechanism of SiO_2_ NPs-mediated drought resistance, RNA sequence libraries of *E*. *macrophylla* seedlings of CK, NPs (treated with 100 mg·L^−1^ SiO_2_ NPs), SD(severe drought), and NPs-SD (100 mg·L^−1^ SiO_2_ NPs + SD) groups were constructed and sequenced. After data filtering, each sample produced an average of 9.895 Gb of clean data. The average base mass of Q30 was above 94.56%, and the GC content was between 43.13% and 44.15%. These parameters indicated a transcriptome sequencing of good quality. By comparing RNA-sequencing data, DEGs were identified among all processing groups (**Supplementary Table 2**).

**Figure 4:**
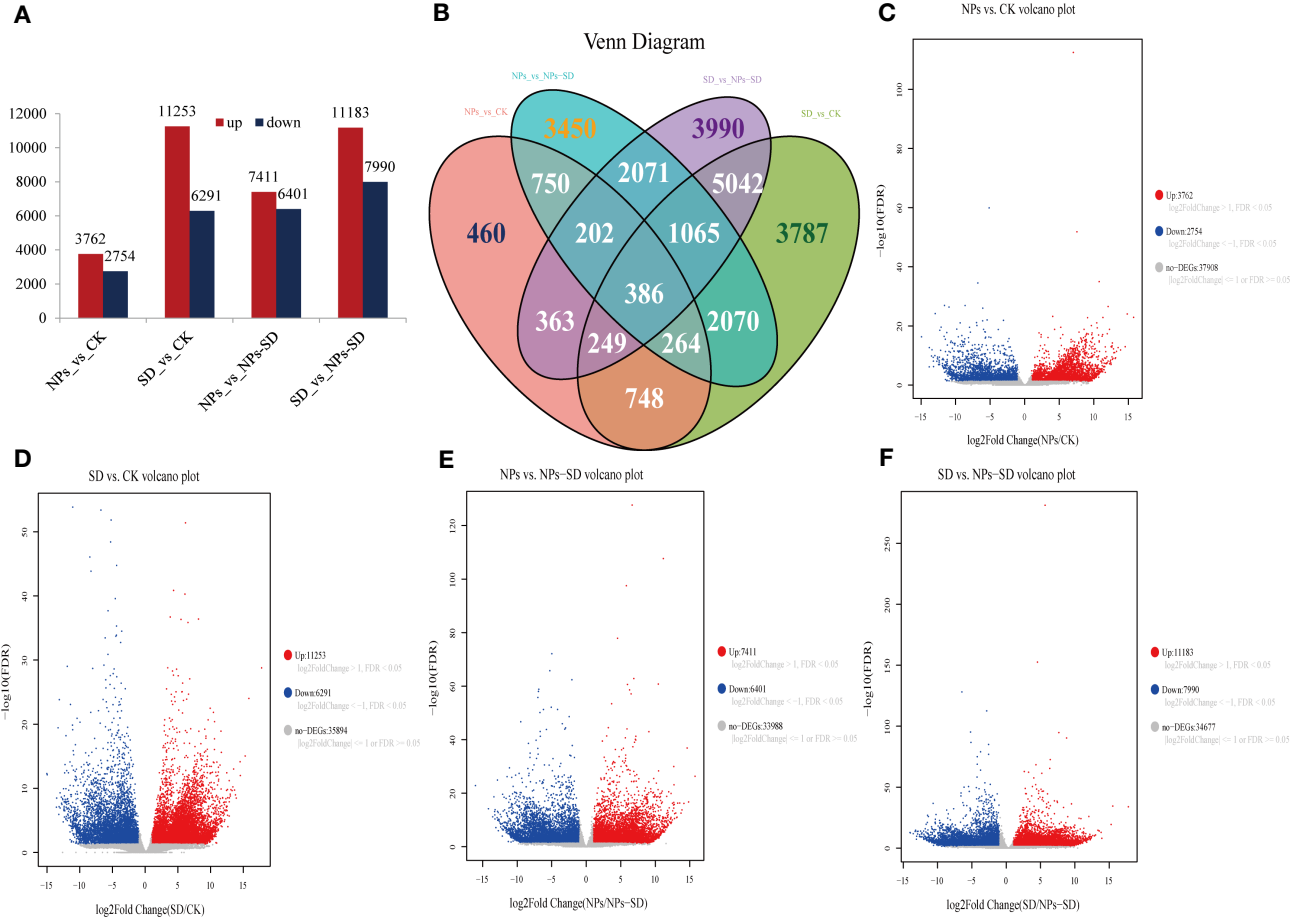
RNA-seq data analysis of leaves in *E. macrophylla* seedlings under different treatments. **(A)** numbers of differentially expressed genes (DEGs) in the NPs_vs_CK, SD_vs_CK, NPs_vs_NPs-SD and SD_vs_NPs-SD; **(B)** venn diagrams of DEGs, the numbers in the graph represent the number of differential genes between treatments, **(C)** volcano plot of NPs_vs_CK, **(D)** volcano plot of SD_vs_CK, **(E)** volcano plot of NPs_vs_NPs-SD, **(F)** volcano plot of SD_vs_NPs-SD. CK: normal watering without SiO_2_ NPs; NPs: normal watering with 100mg·L^−1^SiO_2_ NPs; SD:severe drought treatment without SiO_2_ NPs; NPs-SD: combined severe drought and 100mg·L^−1^SiO_2_ NPs treatment.

Compared with the CK group, a total of 6516 DEGs were identified after NPs treatment, among which 3762 and 2754 were upregulated and downregulated, respectively. A total of 17544 DEGs were identified after SD treatment, among which 11253 and 6291 were upregulated and downregulated, respectively. Compared with the NPs group, a total of 13812 DEGs were identified in the NPs-SD group, among which 7411 and 6401 were upregulated and downregulated, respectively. When compared with the SD group, a total of 19173 DEGs were identified after the NPs-SD group, among which 11183 and 7990 were upregulated and downregulated, respectively (**Figures 4A** **, C–F**; **Supplementary Table 3**). The Venn diagram identified 386 DEGs in four groups (**Figure 4B**). In summary, under drought stress, a large number of genes in *E*. *macrophylla* seedlings changed significantly at the transcriptional level. Furthermore, when SiO_2_ NPs treatment was applied, the gene expression profile of *E*. *macrophylla* seedlings under drought stress may have also changed significantly.

### 3.5 GO function analysis of DEGs

**Figure 5:**
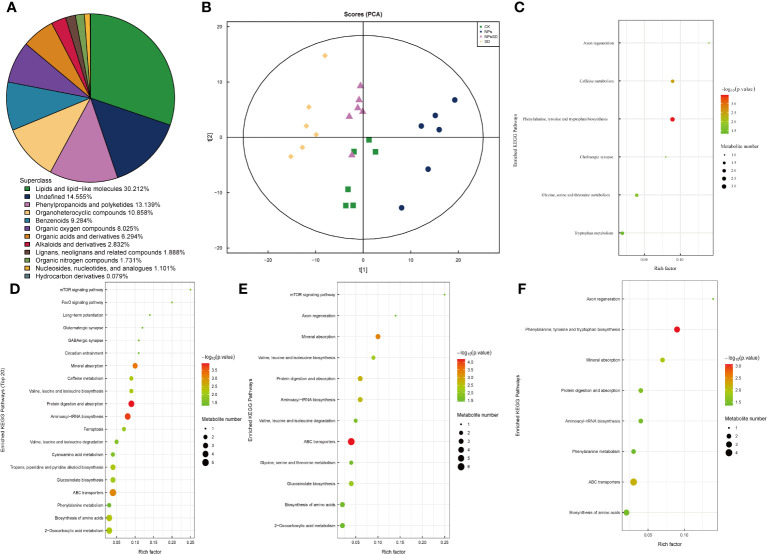
Metabolites analysis of *E*.*macrophylla* seedlings under drought stress. **(A)** species analysis of metabolites, **(B)** PCA analysis of metabolites, KEGG pathway enrichment analysis of DEMs in **(C)** NPs_vs_CK, **(D)** SD_vs_CK, **(E)** NPs_vs_NPs-SD and **(F)** SD_vs_NPs-SD.

Identified DEGs were found to be enriched in 35 pathways (*p*<0.05). The main pathways were related to catalytic activity, binding, defense response, cellular anatomical entity, metabolic process, cellular process, and response to stimulus (**Supplementary Figures 2A–D**). Among them, “pigmentation” was only enriched in the SD_vs_NPs-SD group (**Figure 5D**), and “general transcription initiation factor activity” was only enriched in the NPs_vs_NPs-SD group (**Supplementary Figure 2C**). “Biological adhesion” and “nutrient reservoir activity” were lacked in the treatment group NPs_vs_CK (**Supplementary Figure 2A**), but these pathways were enriched in the other three groups. Among these terms, “defense response” and “response to stimulus” are very important in non-biological stress responses, which are crucial for plants to implement timely defensive measures under drought stress.

### 3.6 Enrichment of DEGs by KEGG metabolic pathway

Under normal water supply condition, DEGs (NPs_vs_CK groups) were mainly concentrated in biosynthesis of secondary metabolites (ko01110), plant hormone signal transduction (ko04075), starch and sucrose metabolism (ko00500), glycolysis/gluconeogenesis (ko00010), and RNA degradation (ko03018) (**Supplementary Figure 3A**). Under normal water supply condition and with no NPs treatment, DEGs (SD_vs._CK groups) were mainly concentrated in ribosome (ko03010), plant hormone signal transduction (ko04075), starch and sucrose metabolism (ko00500), MAPK signaling pathway-plant (ko04016), and carbon fixation in photosynthetic organisms (ko00710) (**Supplementary Figure 3B**). After the treatment with SiO_2_NPs, DEGs (NPs_vs_NPs-SD) were mainly concentrated in the metabolic pathways (ko01100), biosynthesis of secondary metabolites (ko01110), microbial metabolism in diverse environments (ko01120), MAPK signaling pathway-plant (ko04016), and carbon metabolism (ko01200) (**Supplementary Figure 3C**).Under drought conditions, DEGs (SD_vs_NPs-SD) were mainly concentrated in biosynthesis of secondary metabolites (ko01110), ribosome (ko03010), carbon metabolism (ko01200), plant hormone signal transduction (ko04075), and glyoxylate and dicarboxylate metabolism (ko00630) (**Supplementary Figure 3D**).These results indicate that exogenous SiO_2_ NPs treatment has a very important regulatory effect on *E*. *macrophylla* seedlings under drought condition.

### 3.7 qRT-PCR verification of gene expression

To validate RNA-sequencing results, we randomly selected 9 DEGs for real-time fluorescence qPCR analysis. The qPCR expression profiles of all randomly selected genes were consistent with those of the RNA-seq data (**Supplementary Figure 4**), indicating high reliability and high accuracy of RNA sequencing data.

### 3.8 Analysis of differentially expressed metabolites

*E*. *macrophylla* leaf samples (those used for RNA-seq analysis) were subjected to metabolomics (**Supplementary Figure 5**). A total of 1271 types of metabolites were detected, including 838 and 433 positive and negative ion mode metabolites, respectively, and divided into 12 categories (**Figure 5A**; **Supplementary Table 4**). Subsequent principal component analysis showed significant separation among samples (**Figure 5B**). We then compared the trend of relative metabolite content in different treatment groups. For identified DEMs, we employed *p*<0.05 as the threshold for the KEGG pathway enrichment analysis. During the comparison among SD_vs_CK, NPs_vs_NPs-SD, and SD_vs_NPs-SD groups, the following pathways were significantly enriched and co-expressed: biosynthesis of amino acids, ABC transporters, aminoacyl-tRNA biosynthesis, protein digestion, and absorption and mineral absorption (**Figures 5C–F**).

To elucidate the overall trend of the KEGG metabolic pathway, we conducted a differential abundance analysis on the differentially enriched metabolic pathways in four groups (**Supplementary Figure 6**). For NPs_vs_CK, the cholinergic synapse pathway was significantly downregulated, while other pathways were significantly upregulated; in NPs_vs_NPs-SD, the caffeine metabolism pathway was significantly upregulated, while other pathways were significantly downregulated; in SD_vs_CK, glycine, serine and threonine metabolism pathways remained unchanged, while other pathways were significantly upregulated; in SD_vs_NPs-SD, the axon regeneration and phenylalanine metabolism pathways were significantly upregulated and downregulated, respectively.

OPLS-DA was employed to determine differential metabolites and the contribution of each metabolite in the OPLS-DA model was evaluated using VIP. |log2FC|≥1 and VIP≥1 were set as thresholds to filter DEMs. A total of 25 co-existing DEMs were screened in the four groups and their KEGG categories and KEGG enrichment pathway were analyzed (**Supplementary Table 5**). A total of 28 DEMs were successfully classified into the following four categories: lipids and lipid-like molecules (15 DEMs), lignans, neolignans, and related compounds (two DEMs), organoheterocyclic compounds (three DEMs), phenylpropanoids and polyketides (two DEMs), and others (six DEMs). Additionally, to facilitate the observation of the expression of various differential metabolites annotated in the KEGG metabolic pathway, we selected the KEGG metabolic pathway with many differential metabolites more than five, which were mainly concentrated in ko01100 and ko01110 (**Supplementary Table 6**).

### 3.9 Expression of redox-related DEGs

This study leads to the identification of abundant redox-related DEGs, including *SOD*, *POD, LOX, GDH*, and *GST*. In SD_vs_CK, 117 redox-related DEGs (55 upregulated and 62 downregulated, compared with the CK group) were identified; in SD_vs_NPs-SD, 95 redox-related DEGs (49 upregulated and 46 downregulated, compared with the SD group) were identified. A total of 53 genes assigned to the redox process were DEGs in two cases, and these DEGs included seven *CAT* genes, six *SOD* genes, 14 *LOX* genes, three *GDH* genes, and 23 *GST* genes. The SD group had 26 upregulated DEGs, which were downregulated in the NPs-SD group. Further, the SD group had 25 downregulated DEGs, which were upregulated in the NPs-SD group (**Supplementary Figure 7**; **Supplementary Table 7**).

### 3.10 Analysis of transcriptional difference in plant hormone signal transduction and MAPK signaling pathway

The hormone auxin plays an important role in plant abiotic stress. Based on KEGG analysis, we found that a total of 74 and 61 DEGs, related to “the hormone signal transduction,” were enriched in SD_vs_CK and SD_vs_NPs-SD groups, respectively. These genes were mainly involved in the metabolic pathway of GA, JA, CTK, ABA, ETH, IAA, and BR. Furthermore, 39 and 34 DEGs participated in the MAPK signaling pathway-plants pathway in SD_vs_CK and SD_vs_NPs-SD groups, respectively, and these genes were related to BAK1, ETR/ERS, ERF, MYC2, PYR/PYL, and PP2C.

In response to DEGs in the plant hormone signal transduction pathway under drought stress, 54 and 20 Unigenes were downregulated and upregulated, respectively, in SD_vs_CK groups. After SiO_2_ NPs treatment, 45 and 16 Unigenes were downregulated and upregulated, respectively, in SD_vs_NPs-SD groups (**Supplementary Figure 8A**; **Supplementary Table 8**). In response to DEGs in the MAPK signal pathway - plants under drought stress, 22 and 17 Unigenes were downregulated and upregulated, respectively, in SD_vs_CK groups. After SiO_2_ NPs treatment, 13 and 21 Unigenes were downregulated and upregulated, respectively, in SD_vs_NPs-SD groups (**Supplementary Figure 8B**; **Supplementary Table 9**). In summary, the expression of related genes in the plant hormone signal transduction pathway and MAPK signaling pathway – plant pathway may be closely related to the relief of drought stress by treatment with SiO_2_ NPs.

### 3.11 Identification of genes related to fatty acid metabolism and α-linolenic acids metabolism

Under drought conditions, *E*. *macrophylla* seedlings activated the fatty acid metabolism and α-linolenic acid metabolism pathways. In response to DEGs in the fatty acid metabolism pathway under drought stress, 14 and five Unigenes were downregulated and upregulated, respectively, in SD_vs_CK groups. After SiO_2_ NPs treatment, 11 and six Unigenes were downregulated and upregulated, respectively, in SD_vs_NPs-SD groups (**Supplementary Figure 9A**; **Supplementary Table 10**). In response to DEGs in the α -linolenic acid metabolism pathway under drought stress, three and 20 Unigenes were downregulated and upregulated, respectively, in SD_vs_CK groups. After SiO_2_ NPs treatment, eight and 20 Unigenes were downregulated and upregulated, respectively, in SD_vs_NPs-SD groups (**Supplementary Figure 9B**; **Supplementary Table 11**). In summary, DEGs in the fatty acid and α-linolenic acid metabolic pathways may participate in providing relief due to SiO_2_ NPs treatment effect on drought stress.

## 4 Discussion

### 4.1 Physiological mechanism of SiO_2_NPs in reducing drought stress in *E. macrophylla* seedlings

Drought, as one of the main abiotic stress factors, has adverse effects on plant morphology and physiology, thereby slowing down plant growth and development (Liu et al., 2015). This study revealed that drought stress mainly causes the accumulation of MDA and H_2_O_2_ in plants, leading to membrane lipid peroxidation and damaging the structure and function of the cytomembrane. In addition, our experimental results founded that appropriate concentrations of SiO_2_ NPs could effectively alleviate the effects of drought stress on plant growth and development. SiO_2_ NPs, at a concentration of 100 mg·L^−1^, showed the best anti-drought stress effect and greatly improved the drought tolerance of *E*. *macrophylla* seedlings. Mechanistically, SiO_2_ NPs may protect the membrane system (cytomembrane, chloroplast membrane, and thylakoid structure) and reduce the level of damage to the cytomembrane by increasing the activity of antioxidant enzymes and reducing the content of MDA and H_2_O_2_. Conversely, MDA and H_2_O_2_ content peaked and CAT, APX, POD and SOD activity exhibited a significant decrease under at high-level conditions (200 mg·L^−1^ and 500 mg·L^−1^). It may be due to the amage of the antioxidant defense system and the increase of reactive oxygen species (ROS) concentration under the high environmental concentration of SiO_2_ NPs, which has a phytotoxicity effect on the seedlings of *E. macrophylla*. (**Figure 2**). Previously, it was demonstrated that spraying 50 ppm SiO_2_ NP solution under the drought stress could increase the activity of antioxidant enzymes in peas by at least three times, reduce H_2_O_2_ and lipid peroxidation, and improve the dry tolerance of peas (Sutulienė et al., 2022). Another study showed that spraying SiO_2_ NPs increased the activity of CAT, APX, SOD, and GR in strawberries under drought stress, reduced the content of MDA and H_2_O_2_, weakened the oxidative stress response caused by drought, and reduced the harm to strawberries (Zahedi et al., 2020). Akhtar et al. (Akhtar and Ilyas, 2022) found that SiO_2_ NPs significantly increased the activity of antioxidant enzymes (CAT, POD, and SOD) and alleviated lipid peroxidation and oxidative stress induced by drought. In the present study, 100 mg·L^−1^ SiO_2_ NPs alleviated drought stress by regulating the redox response of *E*. *macrophylla* leaves and maintaining cytomembrane function; these results are consistent with those of previous studies.

### 4.2 SiO_2_ NPs alleviate the drought response of *E. macrophylla* seedlings by regulating the expression of antioxidant enzymes

Under environmental stress, such as drought, plants can eliminate redundant ROS by producing antioxidant enzymes (e.g., SOD, CAT, POD, LOX, GDH, and GST) to avoid damage to the cytomembrane (Xu et al., 2023). Under drought stress, 53 genes involved in the POD pathway were differentially expressed; 26 genes were upregulated under SD stress, but downregulated by SiO_2_ NP treatment and 25 genes were downregulated under SD stress, but upregulated by SiO_2_ NP treatment (**Supplementary Figure 7**). In summary, redox-related genes are important regulatory factors for generating the response to drought stress, and SiO_2_ NPs can alleviate oxidative damage by increasing activity of antioxidant enzymes and regulating the expression of redox-related genes.

Drought stress also altered the expression of GSH metabolism-related genes, in which the antioxidant enzyme GST reduces the content of H_2_O_2_ and lipid peroxidation, thereby improving the drought tolerance of plants. The tomato *GST* gene (LeGSTU2) can enhance the drought resistance and salt tolerance of *Arabidopsis thaliana*(Xu et al., 2015). Chen et al. (Chen et al., 2012) reported that atgstu17 is more resistant to drought than the wild-type in the *A. thaliana* mutant. In the present study, 10 GST genes were downregulated in SD_vs_CK groups but significantly upregulated in SD_vs_NPs-SD groups. Hence, SiO_2_ NPs increase GSH activity, reduce lipid peroxidation, and increase the drought tolerance of *E*. *macrophylla* seedlings by increasing the levels of GSH metabolism-related genes.

### 4.3 SiO_2_ NPs improve drought tolerance of *E. macrophylla* seedlings by regulating the hormone signal transduction and MAPK signaling pathway

The MAPK cascade pathway participates not only in plant regulatory mechanisms but also in biotic and abiotic stress responses (Kumar et al., 2020). In the present study, the auxin internal flow vector AUX1 was downregulated under drought stress and controlled by three downregulated genes; however, when NPs treatment was provided, the number of AUX1 downregulated genes was one. Meanwhile, all 9 *AUX/IAA* genes were downregulated under SiO_2_ NPs treatment, which may activate ARF and downstream GH3 to control cell expansion and plant growth under drought stress, thereby improving drought resistance in *E*. *macrophylla* seedlings.

As a hormone that regulates plant growth, ethylene affects a series of developmental processes and stress-resistance reactions in plants. Under the action of Cu^+^, ethylene molecules bind to the ethylene receptor (ETR1), leading to the inactivation of the receptor CTR1 complex (Ji and Guo, 2013). As one of the key elements in the ethylene signal transduction pathway, *ETR* plays a key role in the regulation of growth and development and stress resistance of plants (Bisson and Groth, 2010). EIN3/EIL1 activates the expression of the downstream target gene, ethylene response factor 1 (*ERF1*), at the transcriptional level, which in turn activates the expression of downstream target genes (Zhao and Guo, 2011). *ERFs* are plant-specific and play a trans-acting role in the final step of ethylene signal transduction (Xiao et al., 2013). In the present study, one *ETR* gene, one *SIMKK*, and one *ERF1* gene were upregulated under drought stress after treatment with SiO_2_ NPs (**Supplementary Figure 8A**). Therefore, *ETR, SIMKK*, and *ERF1* may be involved in the drought-response process of *E*. *macrophylla* seedlings mediated by SiO_2_ NPs.

The MAPK cascade is an important defense pathway for plants against abiotic and biotic stress. MAPK regulates the response to extracellular stimuli by transmitting signals. SnRKs are a group of protein kinases that play a role in various physiological activities and can induce the expression of related genes in the ABA signal transduction pathway, which is widely involved in plant adversity resistance (Huai et al., 2008). In the present study, in the SD_vs_CK groups, the overexpression of PYR/PYL in response to drought may have activated PP2C, while the upregulation of gene encoding PP2C may have upregulated the gene encoding the SnRK2 protein, in turn activating the downstream target ABF. SiO_2_ NPs treatment slightly upregulated the *PYR/PYL* gene in *E*. *macrophylla* seedlings under drought stress but significantly downregulated the *PP2C* gene, thereby downregulating the gene encoding SnRK2 protein, inhibiting downstream target ABF and CAT1 activity (**Supplementary Figure 8B**). SiO_2_ NPs slightly reduced the expression of SnRK2, indicating that SiO_2_ NPs can alleviate drought by regulating H_2_O_2_ levels in plants.

### 4.4 SiO_2_ NPs enhance drought tolerance of *E. macrophylla* seedlings by regulating fatty acids and α-linolenic acids

In addition to reducing membrane fluidity and causing physical phase shifts in the membrane lipid, drought stress can directly harm the cytomembrane system. Fatty acids are biosynthesized from acetyl CoA, which has a chain length of C16 or C18 (Parthasarathy et al., 2019). This study indicated that the enzyme-encoding genes involved in the synthesis and metabolism of fatty acids were either up or down-regulated. The α-linolenic acid metabolism and fatty acid oxidation metabolic pathway were activated in *E*. *macrophylla* seedlings under drought stress. Multiple unsaturated fatty acids may be synthesized by the metabolic flow of α-linolenic acids, and downstream gene products; such α-linolenic acids can activate plant defense mechanisms in response to stress and strongly promote the expression of *Gols* (Mata-Pérez et al., 2015). Other plants were also reported to upregulate their lipid metabolism in response to drought stress, which improved the cytomembrane and increased drought tolerance by altering the structural makeup of phospholipids (Hou et al., 2016). Our results exposed that the metabolism of fatty acids was stimulated, with the accumulation of phospholipids accumulated in SD_vs_NPs-SD groups may be explained by the metabolic pathway for α-linolenic acids, which produces a variety of unsaturated fatty acids. The physical phase of the cytomembrane is altered as a result of the upregulation of lipid metabolism, which also increases the drought tolerance of *E. macrophylla* seedlings.

## 5 Conclusions

Physiological, transcriptomic, and metabolomic analyses demonstrated that SiO_2_ NPs can improve the drought tolerance of *E*. *macrophylla* seedlings by multiple mechanisms. Drought stress significantly reduced the content of MDA and H_2_O_2_, and increased the activity of antioxidant enzymes (CAT, APX, POD, SOD). The chloroplast with a loose granular lamellar structure and the mitochondria were also observed to have rupturing of the outer membrane. In particular, SiO_2_ NPs can increase drought resistance by inhibiting the accumulation of MDA and H_2_O_2_ and further enhancing antioxidant enzyme activity. After being subjected to SiO_2_ NPs treatment under drought stress, the chloroplasts are still remained spindle shaped with intact membrane structure, maintaining cell integrity. SiO_2_ NPs controlled the expression of LOX-related genes, other redox-related genes, and the activity of antioxidant enzymes to reduce ROS damage, which improved the drought resistanceof *E. macrophylla* seedlings under drought stress. In addition, SiO_2_ NPs can enhance *E. macrophylla* seedling growth and drought tolerance by controlling important genes in the auxin signal transduction MAPK signaling pathway and the metabolism of fatty acids and α-linolenic acids. This study intuitively revealed the mechanism of SiO_2_ NPs improving plant drought tolerance, and provided theoretical reference for the correct use of SiO_2_ NPs in other species under abiotic stress in the future.

## Data availability statement

The original contributions presented in the study are included in the article/**Supplementary Material**. Further inquiries can be directed to the corresponding author.

## Author contributions

MC: Writing – original draft. S-qJ: Investigation, Methodology, Writing – original draft. SC: Funding acquisition, Visualization, Writing – review & editing. SQ: Data curation, Writing – review & editing. JF: Data curation, Writing – original draft. LX: Investigation, Validation, Writing – review & editing. JS: Writing – review & editing. XG: Writing – review & editing, Data curation. XC: Formal analysis, Writing – review & editing.
