# Supplementary figures and images for: Integrated physiological, transcriptomic, and metabolomic analyses of drought stress alleviation in Ehretia macrophylla Wall. seedlings by SiO2 NPs (silica nanoparticles)
Source: Front Plant Sci. 2024 Feb 2;15:1260140. doi: 10.3389/fpls.2024.1260140 (PMC10869631; doi:10.3389/fpls.2024.1260140)

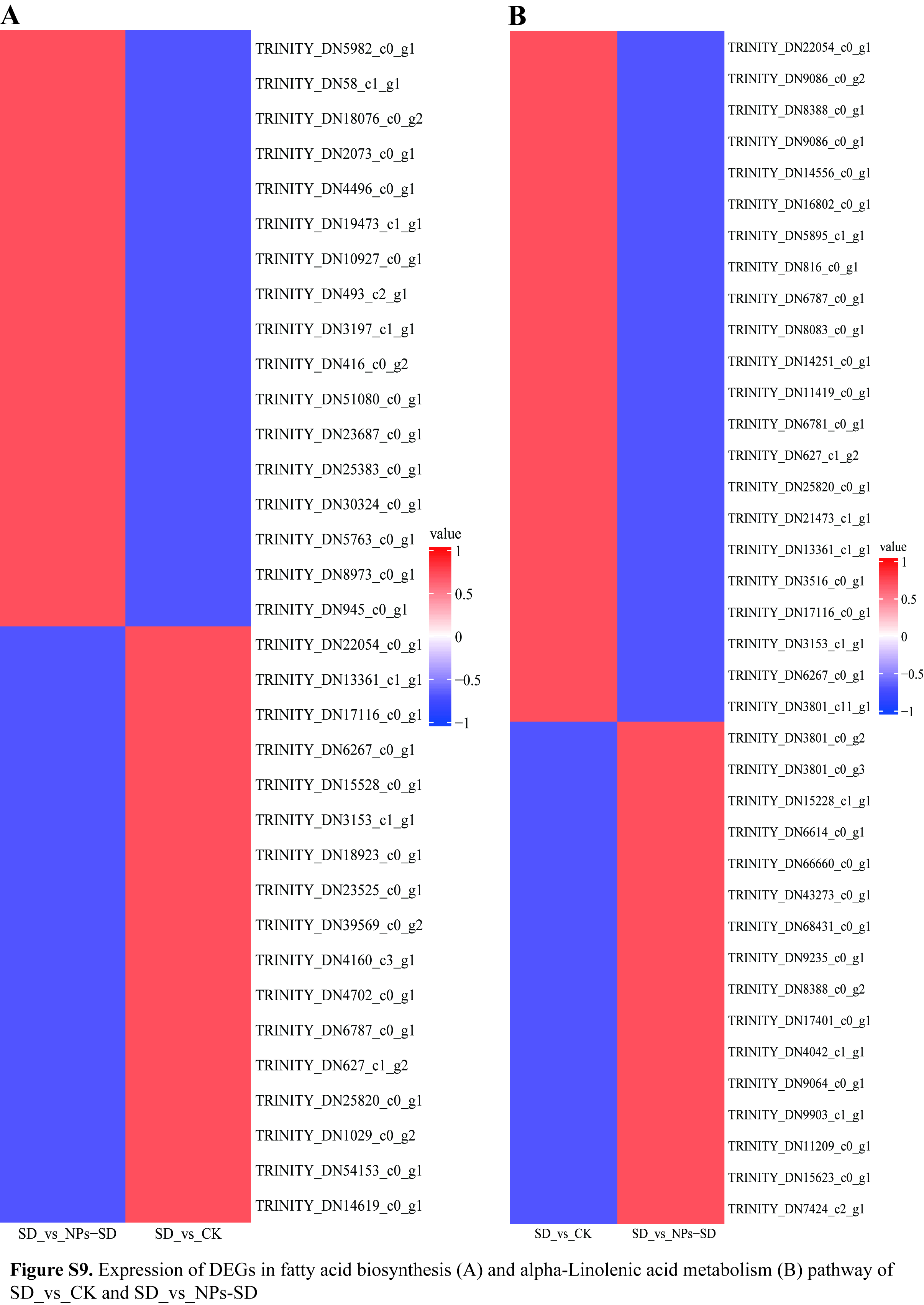

Supplement: Supplementary file 1 [file Image_1.tif]

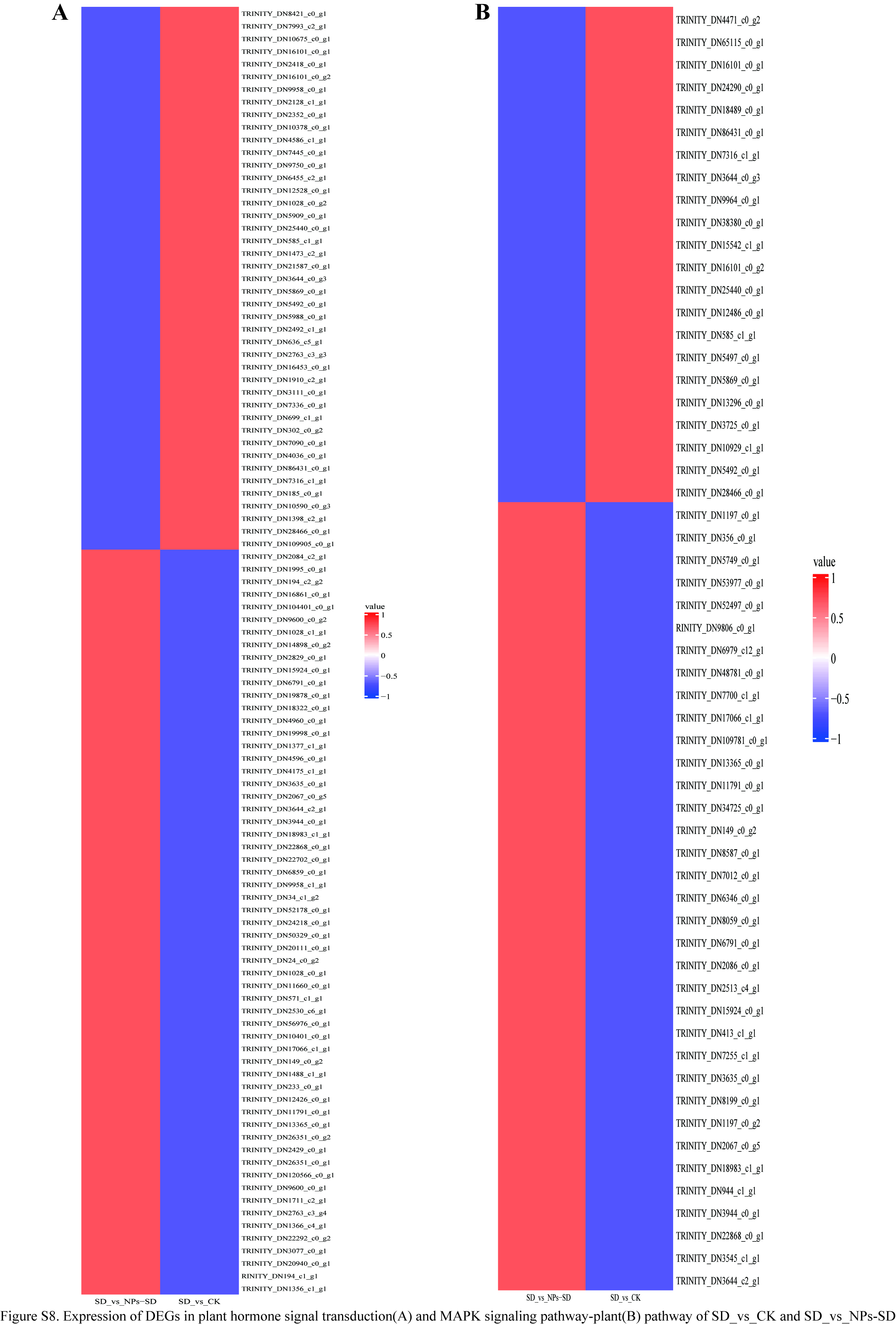

Supplement: Supplementary file 2 [file Image_2.tif]

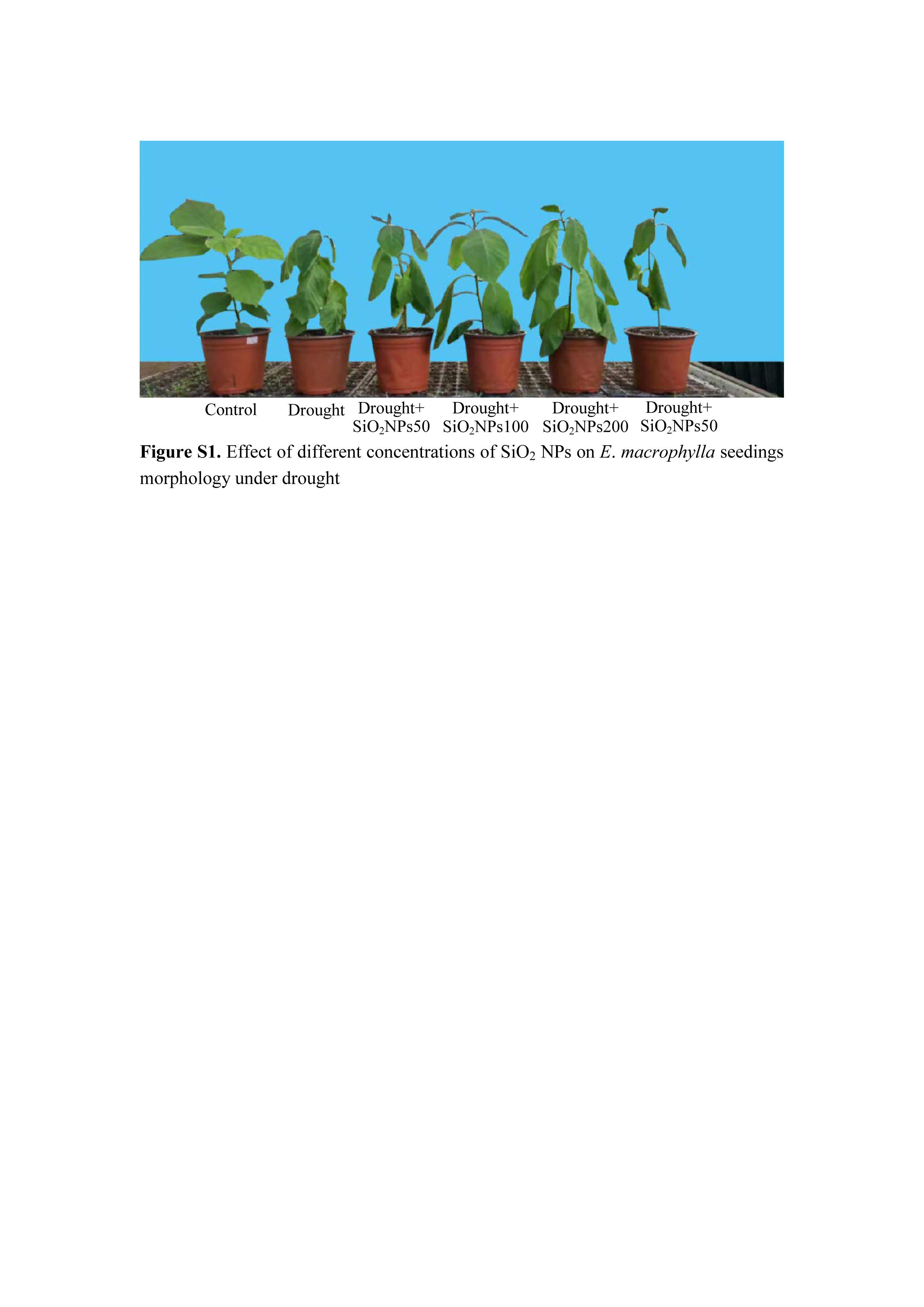

Supplement: Supplementary file 3 [file Image_3.tif]

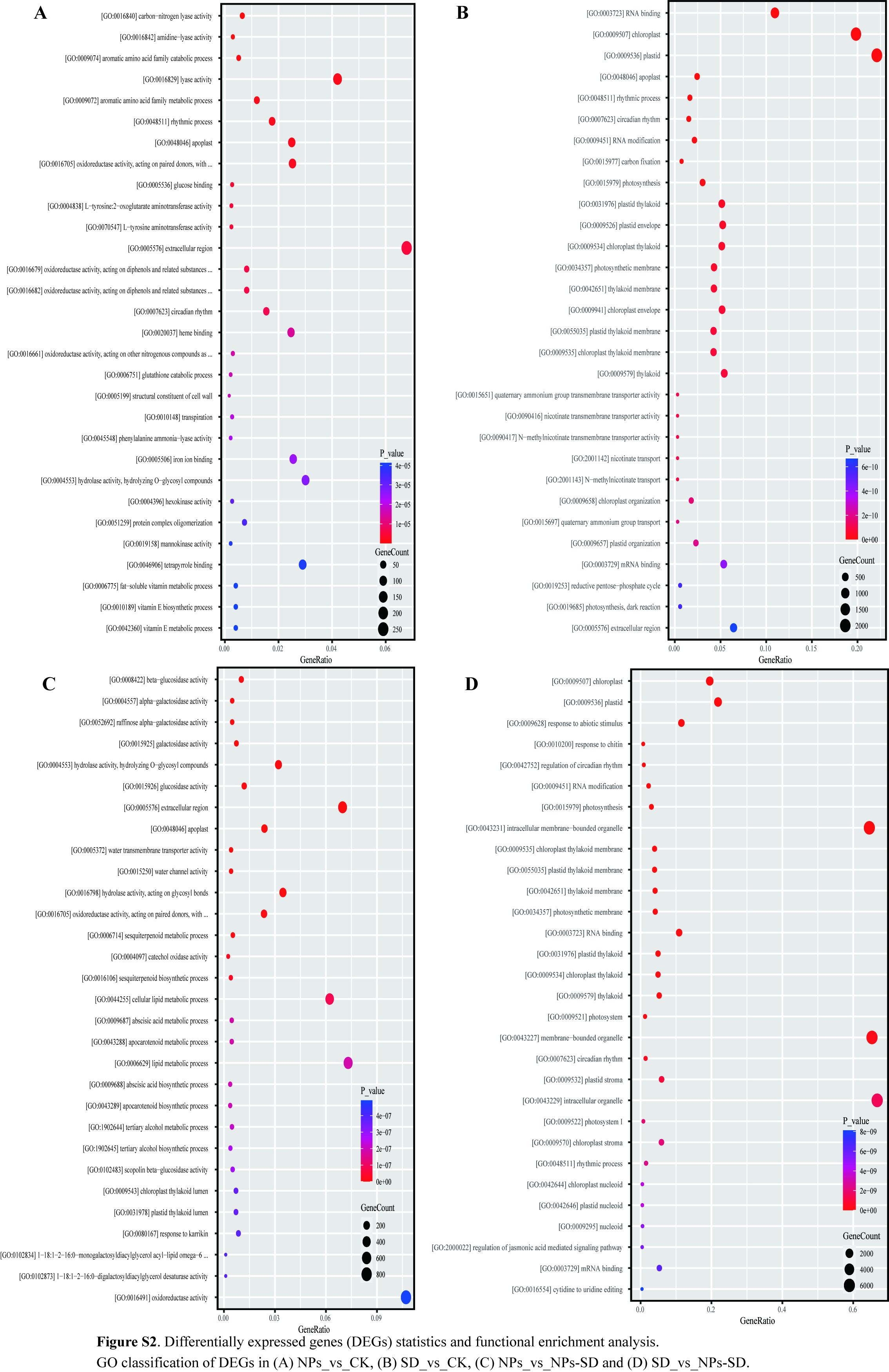

Supplement: Supplementary file 4 [file Image_4.tif]

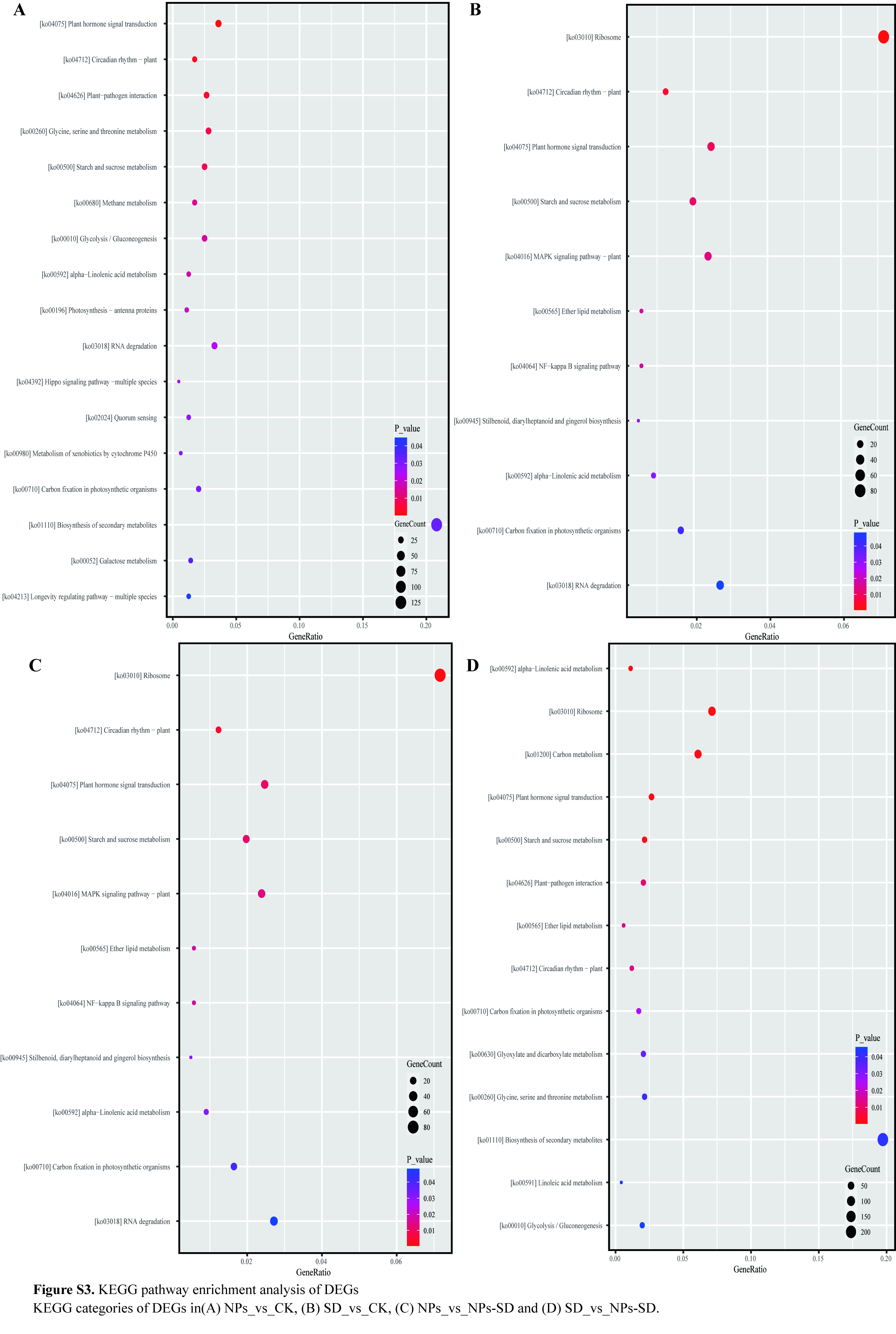

Supplement: Supplementary file 5 [file Image_5.tif]

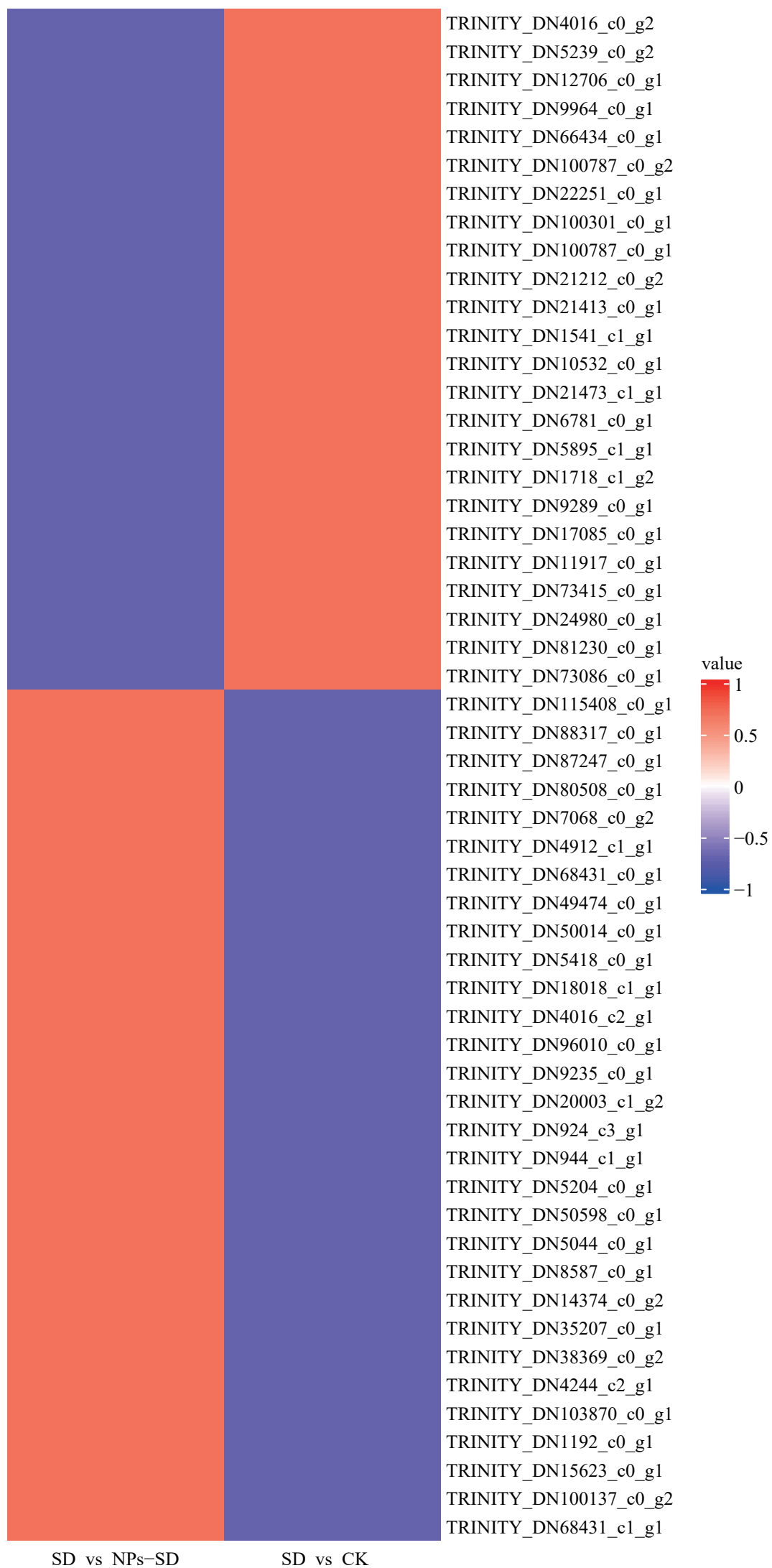

**Figure S7.** Expression of redox-related DEGs of SD\_vs\_CK and SD\_vs\_NPs-SD

Supplement: Supplementary file 9 [file DataSheet_4.pdf]
