# Supplementary material for: Integrated physiological, transcriptomic, and metabolomic analyses of drought stress alleviation in Ehretia macrophylla Wall. seedlings by SiO2 NPs (silica nanoparticles)
Source: Front Plant Sci. 2024 Feb 2;15:1260140. doi: 10.3389/fpls.2024.1260140 (PMC10869631; doi:10.3389/fpls.2024.1260140)
Supplement: Supplementary file 6 [file DataSheet_1.pdf]

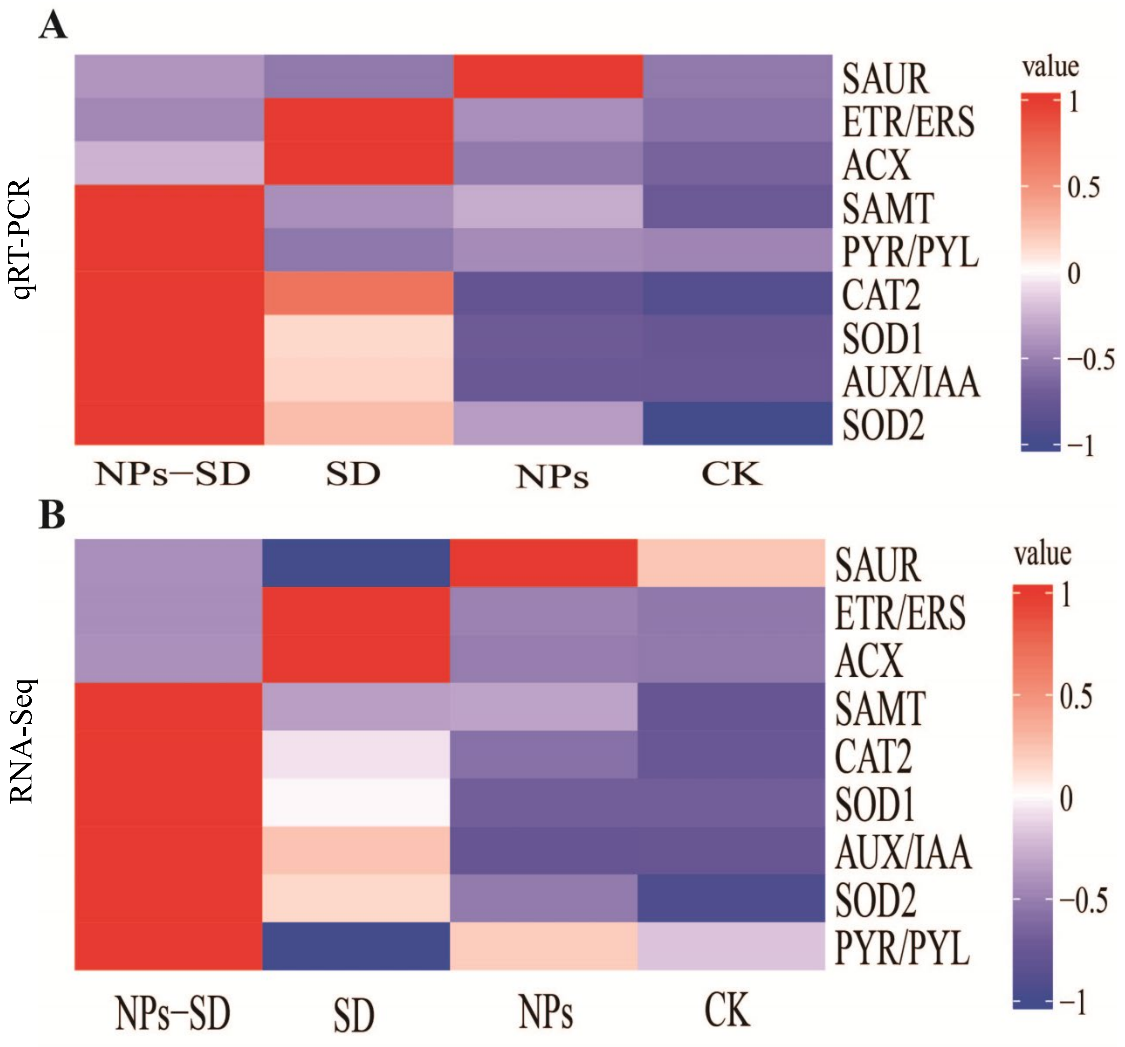

Figure S4. Expression patterns of 9 celected genes identified by RNA-Seq were verified *via* quantitative qRT-PCR. (A),(B) Heatmap showing the expression changes in response to the CK to NPS-SD treatments for each candidate gene, as measured by RNA sequencing(RNA-Seq) and qRT-PCR.
