# Supplementary material for: Integrated physiological, transcriptomic, and metabolomic analyses of drought stress alleviation in Ehretia macrophylla Wall. seedlings by SiO2 NPs (silica nanoparticles)
Source: Front Plant Sci. 2024 Feb 2;15:1260140. doi: 10.3389/fpls.2024.1260140 (PMC10869631; doi:10.3389/fpls.2024.1260140)
Supplement: Supplementary file 7 [file DataSheet_2.pdf]

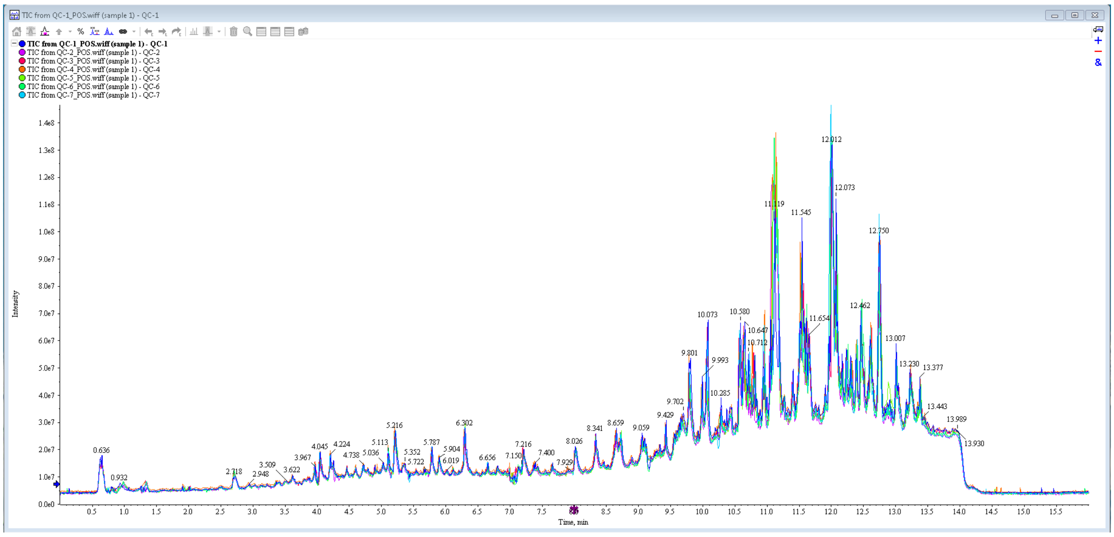

Figure S5a Positive ion mode QC sample total ion chromatograms

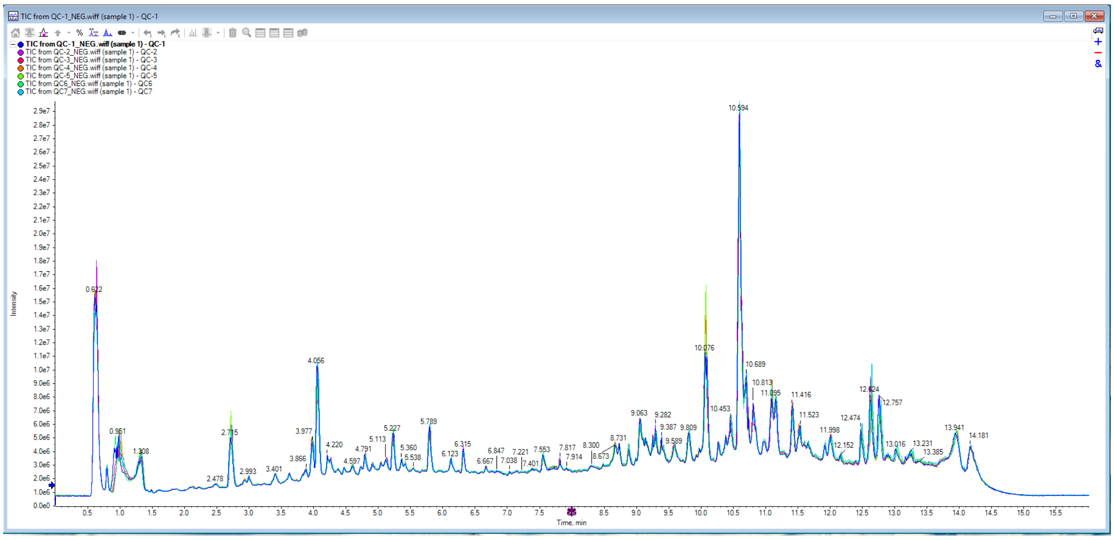

Figure S5b Negative ion mode QC sample total ion chromatograms
