# Supplementary material for: Integrated physiological, transcriptomic, and metabolomic analyses of drought stress alleviation in Ehretia macrophylla Wall. seedlings by SiO2 NPs (silica nanoparticles)
Source: Front Plant Sci. 2024 Feb 2;15:1260140. doi: 10.3389/fpls.2024.1260140 (PMC10869631; doi:10.3389/fpls.2024.1260140)
Supplement: Supplementary file 8 [file DataSheet_3.pdf]

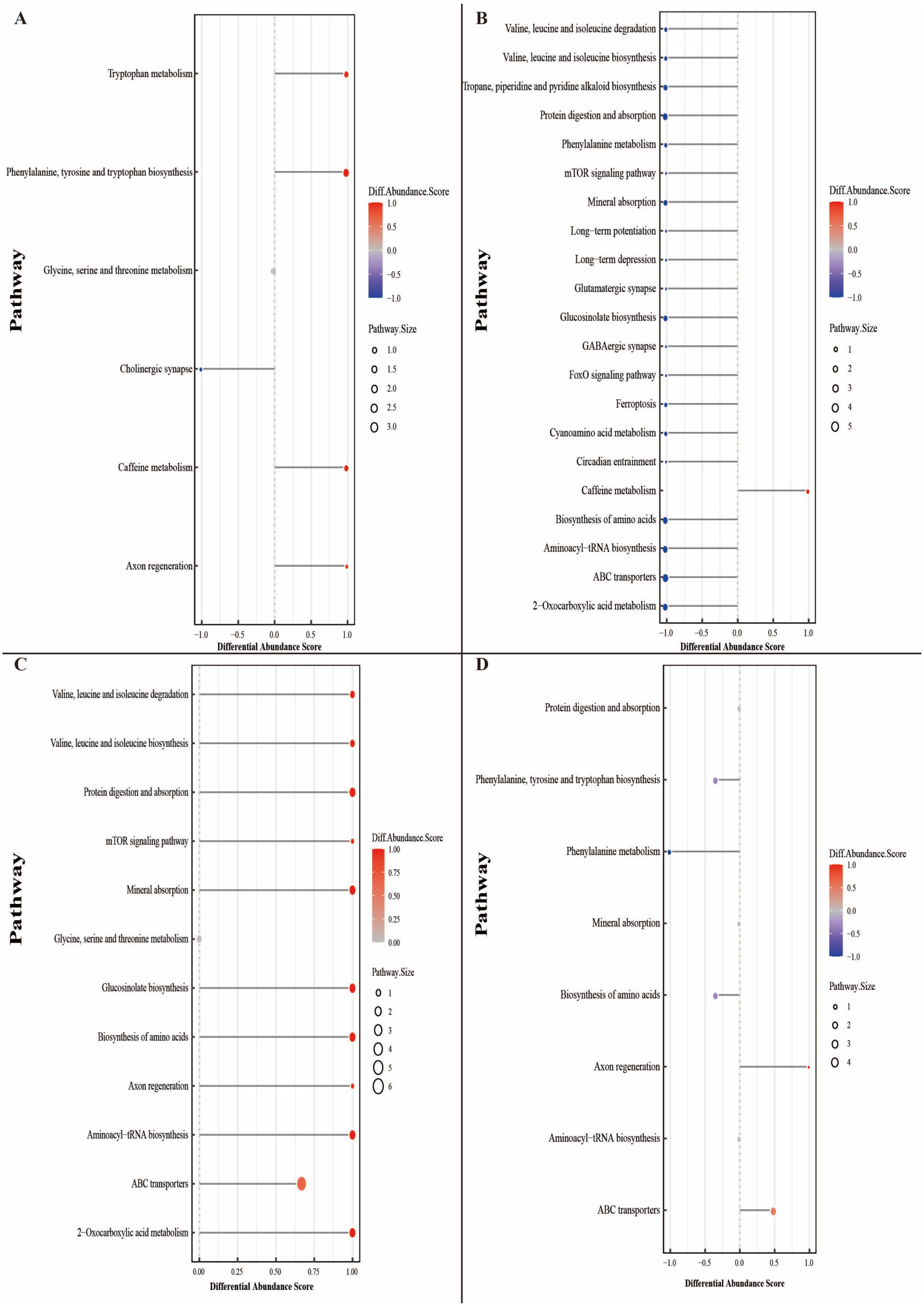

Figure S6. KEGG pathway differential abundance analysis of DEMs Differential abundance analysis of DEMs in (A) NPs_vs_CK, (B) SD_vs_CK, (C) NPs_vs_NPs-SD and (D) SD_vs_NPs-SD.
